# The Role of Autophagy in Acute Myocardial Infarction

**DOI:** 10.3389/fphar.2019.00551

**Published:** 2019-05-31

**Authors:** Du Wu, Kangfeng Zhang, Pengfei Hu

**Affiliations:** ^1^ Department of Internal Medicine, The WuYun Mountain Sanatorium of Hangzhou, Hangzhou, China; ^2^ Department of Cardiology, The Second Affiliated Hospital of Zhejiang Chinese Medical University, Hangzhou, China

**Keywords:** acute myocardial infarction, autophagy, cardiomyocyte, ischemia, reperfusion

## Abstract

Acute myocardial infarction refers to a sudden death of cardiomyocytes, which leads to a large mortality worldwide. To attenuate acute myocardial infarction, strategies should be made to increase cardiomyocyte survival, improve postinfarcted cardiac function, and reverse the process of cardiac remodeling. Autophagy, a pivotal cellular response, has been widely studied and is known to be involved in various kinds of diseases. In the recent few years, the role of autophagy in diseases has been drawn increasing attention to by researchers. Here in this review, we mainly focus on the discussion of the effect of autophagy on the pathogenesis and progression of acute myocardial infarction under ischemic and ischemia/reperfusion injuries. Furthermore, several popular therapeutic agents and strategies taking advantage of autophagy will be described.

## Introduction

Acute myocardial infarction, also well known as acute heart attack, is induced by the sudden blockade or occlusion of a major branch of a coronary artery, thus leading to the ischemia or infarct of cardiomyocytes (Stanley, 2001; Klopsch et al., 2019; Musher et al., 2019). So far, it is generally acknowledged that at 12 h or a little later after the onset of irreversible ischemia, the earliest change is observed morphologically as pallor of the myocardium, referring to the ischemia of cardiomyocytes (Burke and Virmani, 2007; Li et al., 2019; Shoji et al., 2019). The disturbance of blood flow leads to the deprivation of energy supply, which results in the dysfunction and death of cardiomyocytes (Burke and Virmani, 2007). The cardiac damage of acute myocardial infarction is widely acknowledged *via* the ischemic and ischemia/reperfusion injuries, thus resulting in the detrimental effect on cardiomyocytes as well as cardiac functions. The mechanisms of myocardial injury under irreversible ischemic stress included increased cytosolic Ca2^+^ induced by the inhibition of Na^+^, K^+^-ATPase and disturbance of mitochondria, leading to the activation of various kinds of proteases, cleavage of anchoring cytoskeletal proteins, and progressive increases in cell membrane permeability (Jennings and Ganote, 1974; Jennings et al., 1995; Stanley, 2001; Buja, 2005; Burke and Virmani, 2007). Consequently, protecting cardiomyocytes against ischemic injury serves as a vital strategy for the treatment of acute myocardial infarction. Furthermore, the infiltration of inflammatory cells such as macrophages and neutrophils has been observed in the border areas on the occurrence of acute myocardial infarction, indicating that suppression of inflammatory reaction in cardiac ischemic regions also provides a potential and effective pathway (Crea and Libby, 2017; Loyer et al., 2018; Ong et al., 2018; Zhang et al., 2018a; Peng et al., 2019). So far, although increasing knowledge has been gained on acute myocardial infarction and various kinds of interventions have been developed, the gross mortality of acute myocardial infarction patients remains high, and more effective therapeutic strategies are still demanded.

Autophagy is a vital metabolic process for the degradation of senescent or damaged proteins and organelles into amino acids and fatty acids for energy production and recycling (Catana et al., 2018; Wang et al., 2018c). It is activated in response to nutrient starvation or metabolic stress for the maintenance of tissue functions and homeostasis (Dong et al., 2018). It has been demonstrated that basal autophagy is vital for the maintenance of normal cardiac functions (Zhang et al., 2018b). Under the ischemic stress, autophagy is activated to protect cardiomyocytes against ischemic or ischemia/reperfusion injury (Sciarretta et al., 2014; Wang et al., 2018b). Furthermore, autophagy can act as an inflammatory suppressor, thus contributing to the alleviation of progress of cardiac injury (Mohajeri and Sahebkar, 2018; Ryter et al., 2018; Li et al., 2018c). However, excessive activation of autophagy may lead to a detrimental effect on the heart in the reperfusion damage as well as other stress conditions, indicating the controversial effect of autophagy in cardiac ischemia (Ma et al., 2011; Bai et al., 2018). Here in this paper, we will discuss the role of autophagy in acute myocardial infarction in the alleviation of myocardial infarction under the ischemic and ischemia/reperfusion injuries. Furthermore, several potential and effective therapeutic strategies taking advantage of autophagy will be discussed, aiming to provide insights in the development of new drugs or therapies against acute myocardial infarction.

## Biology of Autophagy

The word “autophagy,” derived from Greek roots “auto” (self) and “phagy” (eat), was initially created by Dr. Christian de Duve in 1960s, referring to a cellular catabolic process in which intracellular substances were degraded by itself (Klionsky et al., 2016; Ktistakis, 2017). Recently, Dr. Yoshinori Ohsumi was awarded the 2016 Nobel Prize in Medicine or Physiology for his discovery of cellular autophagy processes, which made a big step in the development of novel therapies for various kinds of diseases taking advantage of autophagy (Van Noorden and Ledford, 2016; Harnett et al., 2017). So far, it has been widely acknowledged that autophagy is a vital catabolic mechanism relying on lysosomes (Hewitt and Korolchuk, 2017). During the autophagy process, some long-lived or misfolded proteins as well as damaged organelles are transferred into lysosomes for degradation into fundamental nutrient substance such as amino acids for recycling and further use (Boya et al., 2018; Li et al., 2018b). According to the patterns of cargo delivery to the lysosomal lumen and physiological functions, autophagy has been mainly classified into three types, namely macroautophagy, microautophagy, and chaperone-mediated autophagy (Zhang et al., 2018c). Macroautophagy is a catabolic process characterized by sequestration of cytoplasmic material in double membrane vacuoles called autophagosomes, which are then delivered to the lysosome for degradation (Wang et al., 2018c). Microautophagy is a non-selective lysosomal degradative process referring to the engulfment of cytoplasmic constituents through invagination of the lysosomal/vacuolar membranes (Kalachev and Yurchenko, 2017). Chaperone-mediated autophagy is a type of autophagy that allows the degradation of cytosolic proteins depending on chaperones. It is recognized as the only autophagy process that allows selective degradation of soluble cytosolic proteins in lysosomes (Alfaro et al., 2018). In addition to those three kinds of classic autophagy, some special forms of autophagy (selective autophagy) have been described, including mitophagy, pexophagy, ribophagy, xenophagy, and secretory autophagy (Ponpuak et al., 2015; Mao and Klionsky, 2017; An and Harper, 2018; Broda et al., 2018; Tsuchiya et al., 2018). Since macroautophagy is the most extensively studied form of autophagy, here in this review, the role of macroautophagy in acute myocardial infarction will be discussed (hereafter referred to as “autophagy”).

Autophagy process is an evolutionarily conserved process from yeast to mammals (Schultz et al., 2017). So far, more than 30 kinds of autophagy-related genes (Atgs) proteins are recognized to be involved in the process of autophagy (Diaz et al., 2017; Wildenberg et al., 2017). Generally speaking, autophagy is performed in two major steps (Bento et al., 2016; Shao et al., 2016; Zachari and Ganley, 2017; Wang et al., 2018c). In the first step, the Unc-51-like kinase 1 (ULK1), focal adhesion kinase family interacting protein of 200 kD (FIP200), Atg13, and Atg101 are combined to form the Atg1 complex, which subsequently triggers the assembly of Beclin-1, Atg14, VSP15, and VSP34 comprising the Class III phosphatidylinositol 3-hydroxy kinase (PI3K) complex. The Class III PI3K complex leads to the membrane nucleation process and formation of the cup-shaped, lipid bilayer membrane-structured phagophore. Further membrane expansion and fusion together with the Atg5-Atg12-Atg16L1 and light chain 3 (LC3) result in the occurrence of the intracellular, spherical double-membraned autophagosomes enclosing proteins and organelles. In the second step, with the disposal of “coat proteins” (LC3-II) on the surface, autophagosomes integrate with lysosomes to form the single membrane-structured autolysosomes, the functional units of autophagy for degradation and recycling.

So far, among the whole complicated signaling network of autophagy, two classical signaling pathways have been described for the inhibitory and promoted regulation of autophagy (Inoki et al., 2012; Shao et al., 2016). The Class I PI3K-mammalian target of rapamycin (mTOR) signaling pathway, a classical inhibitory pathway, is triggered in the presence of nutrient enrichment, to stimulate the activation of mTOR and the mTOR complex (mTORC1) *via* protein kinase B (Akt) pathway, thus inhibiting the formation of the Atg1 complex (Kaur and Sharma, 2017; Perez-Alvarez et al., 2018). The other classical signaling pathway of autophagy is induced by AMP-activated protein kinase (AMPK), a sensor of stress and nutrient input, which promotes the occurrence of autophagy process through activating the ULK1 kinase complex by inactivating mTORC1 or phosphorylating ULK1 at various serine residues (Dodson et al., 2013; Zhao et al., 2018a). So far, several kinds of agents have been developed for the blockade or induction of autophagy in different mechanisms, such as rapamycin, chloroquine, bafilomycin A1, and 3-methyladenine (3-MA), thus largely facilitating the fundamental study of autophagy (Germic et al., 2017; Bhat et al., 2018; Zhao et al., 2018b).

It has been widely reported that the induction of autophagy in a moderate extent plays a protective role in organisms. For instance, autophagy has been demonstrated to inhibit apoptosis in various kinds of cells (Pott et al., 2018; Wang et al., 2018a). It has also been reported that autophagy contributes to the suppression of inflammatory and immune reaction in many kinds of inflammation-related disorders (Burger et al., 2018; Pankratz et al., 2018; Chen et al., 2019; Gogiraju et al., 2019). Autophagy has been shown to be vital in the inhibition of the pathogenesis and progression of various kinds of diseases in the central nervous system (ischemic stroke, multiple sclerosis, Alzheimer’s disease), cardiovascular system (myocardial infarction, heart failure, atherosclerosis), endocrine system (diabetes, obesity), digestive system (inflammatory bowel disease), and so on (Crino, 2016; Cosin-Roger et al., 2017; Schwerd et al., 2017; Zhao et al., 2017; Lambelet et al., 2018; Liu et al., 2018c; Sciarretta et al., 2018). However, it has also been noted that overinduction of the autophagy process might lead to the detrimental effect of cells, the so-called “autophagic cellular death” in organisms, indicating the importance of controlling the extent of autophagy induction in the treatment of diseases (Lambelet et al., 2018; Wang et al., 2018c).

## Autophagy in Acute Myocardial Infarction

From our previous description, the acute myocardial infarction leads to the infarct or death of cardiomyocytes under the stress of ischemic or ischemia/reperfusion injury. As a result, to fight against acute myocardial infarction, strategies should be developed to protect cardiomyocytes against such injuries. In this section, the role of autophagy in cardioprotection will be discussed in the challenge of ischemic or ischemia/reperfusion injury. However, it has been demonstrated that overactivation of cardiac autophagy leads to a detrimental effect of acute myocardial infarction, which will also be discussed in the following contents.

### Cardiac Autophagy in Ischemic Injury

#### Beneficial Effect of Cardiac Autophagy in Ischemic Injury

As we discussed above, autophagy is vital for the maintenance of cellular function and homeostasis for its degradation of long-lived proteins and damaged organelles to prevent protein aggregate accumulation to cytotoxic levels. Baseline autophagy or “adaptive” induction of autophagy produced a protective and alleviative role in ischemic injury.

Baseline autophagy is necessary in maintaining cardiac structure and function since impaired autophagy has been reported to contribute to the pathogenesis and progression of heart failure (Pattison and Robbins, 2008; Bhuiyan et al., 2013). For instance, it was demonstrated that depletion of Atg7 significantly increased the pathology in cardiomyocytes (Pattison et al., 2011). Ulk1-deleted mice showed exacerbation of lipotoxicity associated with retarded cardiac function and FIP200 and Atg13 were proven to be necessary for cardiac development during embryogenesis (Gan et al., 2006; Kaizuka and Mizushima, 2016; Ghosh and Pattison, 2018). Furthermore, it was demonstrated that genetic variants on chromosome 1p13.3 near the damage-regulated autophagy modulator 2 (DRAM2) gene were associated with non-ST elevation myocardial infarction in a case–control study (Salo et al., 2015). Those findings indicate the importance of autophagy in cardiac tissue. Autophagy was previously reported to be upregulated in patients with coronary artery disease or acute myocardial infarction compared to the healthy controls (Bullon et al., 2017; Demircan et al., 2018). In addition, it was demonstrated that the declination of autophagy and mitochondrial impairment led to impaired host response to hypoxic-ischemic injury, thus producing the detrimental effect of cardiomyocytes (Ham and Raju, 2017). In patients or animals with diabetes, hyperglycemia, or other metabolic derangements, cardiac function was detrimental because of the dysfunction of cardiac autophagy, indicating a future potential and effective therapeutic strategy in preserving cellular homeostasis and survival in patients with metabolic derangements (Baranyai et al., 2015; Sciarretta et al., 2015). Furthermore, mitophagy, a special form of autophagy functioning in the maintenance of mitochondrial homeostasis, has been considered to play as a cardioprotective under ischemic injury, indicating the important role of mitophagy in ischemic injury (Tahrir et al., 2019).

So far, autophagy has been widely reported to produce an alleviative effect in acute myocardial infarction. Some researchers reported that the “adaptive” induction of autophagy functioned in attenuation of aggregate/aggresome formation in heart, thus suppressing the detrimental effect of protein aggregation (Tannous et al., 2008). In addition, Aisa et al. (2017) reported that autophagy could reduce the infarct size of acute myocardial infarction after left anterior descending ligation in rat models. Similar conclusion was drawn by Kanamori et al. (2011), who showed that the administration of autophagy inhibitor, bafilomycin A1, significantly increased the infarct size of animal acute myocardial infarction models, indicating autophagy as an innate and potent process that produced a cardioprotective effect against ischemic injury during acute myocardial infarction. It was demonstrated that the autophagy process was upregulated through the AMPK-mTOR signaling pathway in cardiomyocytes, thus leading to the attenuation of acute myocardial infarction in animal models (Li et al., 2016, 2017; Foglio et al., 2017). It was also shown by Sciarretta et al. (2012) that genetic inhibition of AMPK signaling pathway led to the dysfunction of autophagy process, which resulted in the increase in infarct size in acute myocardial infarction. The phosphatase and tensin homolog deleted on chromosome 10 (PTEN)-PI3K-Akt signaling pathway was also demonstrated to be involved in the induction of cardiac autophagy in *in vitro* hypoxia (Zhang et al., 2017). Furthermore, Wu et al. (2014, 2017) demonstrated that upregulation of autophagy flux could protect cardiomyocytes against ischemia and attenuate adverse cardiac remodeling after acute myocardial infarction in rat models. The administration of autophagy inhibitor, 3-MA, contributed to adverse cardiac remodeling through the induction of nuclear factor-κB (NF-κB) activation in animal acute myocardial infarction models (Wu et al., 2014).

#### Detrimental Effect of Cardiac Autophagy in Ischemic Injury

As discussed above, baseline autophagy or autophagy induced in a proper extent produces a protective effect in ischemic injury through the maintenance of cellular homeostasis and degradation of organelles or misfolded proteins for ATP production in cardiomyocytes. However, it has been reported that under the condition of severe ischemia, the overwhelming induction of cardiac autophagy may promote cell death and worsen cardiac performance (Li et al., 2018a; Liu et al., 2018a; Xiao et al., 2018).

According to a previous study conducted by Lu et al. (2018), extensively induced autophagy was revealed to be detrimental in the severity of acute myocardial infarction in animal models, and exercise preconditioning was reported to reduce the high level of serum cTnI and severity of myocardial ischemia/hypoxia through the downregulation of excessive autophagy and cardiac K_ATP_ channels. In addition, transforming growth factor-β receptor I downregulation induced by loss of Sirt7, a kind of factor in response to acute myocardial infarction, was blocked by autophagy inhibitor, indicating that Sirt7 could maintain transforming growth factor receptor I *via* modulating autophagy in tissue repair process in response to ischemic injury (Araki et al., 2015).

In the occurrence of cardiac ischemia or infarction, hypoxia-induced injury serves as one of the major factors in cardiac damage through the induction of apoptosis and excessive autophagy process (Zhang et al., 2016). It has been demonstrated that the exosome-transported miRNA-93-5p produced a cardioprotective effect in the animal model of acute myocardial infarction as well as in an *in vitro* model of hypoxic H9C2 cells through the suppression of hypoxia-induced autophagy and inflammatory cytokine expression by targeting Atg7, a vital autophagy-related gene and Toll-like receptor 4 (Liu et al., 2018b). In addition, another microRNA, MicroRNA-223, was reported to protect neonatal rat cardiomyocytes and H9C2 cells from hypoxia-induced apoptosis and excessive autophagy through the Akt/mTOR pathway by targeting PARP-1 (Liu et al., 2018c). Taken together, those previous studies demonstrated the detrimental effect of hypoxia-induced excessive autophagy in the severity of acute myocardial infarction.

### Cardiac Autophagy in Ischemia/Reperfusion Injury

#### Beneficial Effect of Cardiac Autophagy in Ischemia/Reperfusion Injury

As we discussed above, the “adaptive” induction of autophagy process, which is responsible for the degradation and recycling of proteins and organelles, is vital for the maintenance of cellular function under certain stress conditions. In myocardial ischemia/reperfusion, induction of autophagy in an adaptive manner contributes to the alleviation of cardiac damage under ischemia/reperfusion injury.

For instance, a positive association between pharmacological upregulation of autophagy and increased resistance to myocardial ischemia/reperfusion injury was demonstrated by Przyklenk et al. (2011) in an *in vivo* swine model of acute myocardial infarction, despite the fact that the induction of autophagy was either protective or detrimental of the severity of acute myocardial infarction in patients. Similar to the effect on cardiac ischemic injury, baseline autophagy produces a cardioprotective effect against ischemia/reperfusion injury. It was demonstrated that the impairment of autophagosome clearance mediated in part by reactive oxygen species-induced decline in lysosome-associated membrane protein-2 and upregulation of Beclin-1 under ischemia/reperfusion injury contributed to the enhancement of cardiomyocyte death (Ma et al., 2012). In addition, it was demonstrated that the quality control of mitophagy served as an effective pathway in the protective cardiomyocytes under ischemia/reperfusion injury *via* the maintenance of mitochondrial homoeostasis (Siasos et al., 2018). Those findings indicated that the restoration of baseline autophagy could serve as an effective and potential strategy in fighting against ischemia/reperfusion injury.

In addition, the protective effect of autophagy in cardiomyocytes against ischemia/reperfusion injury was also reported by several other researchers, indicating the alleviative role of autophagy in acute myocardial infarction (Sala-Mercado et al., 2010; Sengupta et al., 2011; DuSablon et al., 2017; Song et al., 2017; Fu et al., 2018). It was reported that the natural compound of visnagin delivered by nanoparticles induced cardioprotection, reducing the size of the acute myocardial infarction and ameliorating cardiac dysfunction through the induction of autophagy and thus leading to the inhibition of apoptosis process under ischemia/reperfusion injury (Fu et al., 2018). The proper induction of autophagy process could largely improve cell viability, contributing to the protection of cardiomyocytes (DuSablon et al., 2017). Taken together, those studies demonstrated the cardioprotective effect of autophagy induction in the prevention of ischemia/reperfusion damage.

#### Detrimental Effect of Cardiac Autophagy in Ischemia/Reperfusion Injury

As discussed above, baseline autophagy or adaptively induced cardiac autophagy plays a cardioprotective role under ischemia/reperfusion injury. However, attention should be paid to the development of therapeutic strategies against acute myocardial infarction taking advantage of upregulation of autophagy against ischemia/reperfusion injury. The autophagy process induced by several factors has been reported to be detrimental to cardiomyocytes.

For example, it was reported that ischemia/reperfusion-induced autophagy could lead to the cascade induction of apoptosis, necrosis, and inflammatory reaction, which led to the damage of cardiac cell viability (Qian et al., 2009). In addition, the NF-κB-induced autophagy was demonstrated to exacerbate myocardial injury in acute myocardial infarction, indicating the importance of the source of autophagy induction (Zeng et al., 2013, 2016). Furthermore, the excessive autophagy process induced by hypoxia was reported to lead to cardiac cell viability, which was reported to be possible to involve the PI3K/AKT/mTOR pathway (Qin et al., 2018).

In some conditions, the role of autophagy in acute myocardial infarction in the process of ischemia and reperfusion is controversial. For example, it was previously reported that mitochondrial aldehyde dehydrogenase (ALDH2), a kind of enzyme that catalyzes the oxidation of aldehydes, could significantly promote autophagy process during ischemia *via* the activation of AMPK and downregulation of mTOR, thus producing a cardioprotective effect. On the contrary, during the reperfusion process, ALDH2 could suppress the level of the autophagy process through the activation of Akt and mTOR, thus protecting cardiomyocytes against cell death in hypoxia and reoxygenation (Ma et al., 2011). Those findings indicated that attention should be paid to the development of therapies against acute myocardial infarction particularly for their potential controversial effects on ischemic and reperfusion conditions.

#### Pharmacological Intervention of Autophagy in the Treatment of Acute Myocardial Infarction

So far, an increasing number of fundamental and clinical studies have been conducted in the development of therapeutic strategies taking advantage of autophagy in the treatment of acute myocardial infarction. Fortunately, numerous promising autophagy inducers have been described, several of which will be briefly described below together with their pharmacological mechanisms (summarized in Table 1).

**Table 1:** Potential mechanisms of autophagy inducers in the treatment of acute myocardial infarction.

Autophagy inducer		Potential mechanisms	Reference
Apoptosis inhibitor	Apelin	Activation of Apelin/APJ system	Liu et al., 2017
	Tongxinluo	Inducing AMPK-mediated autophagy	Li et al., 2017
	CREG	Regulating lysosomal protein transfer	Song et al., 2017
	Atorvastatin	Activating AMPK-mTOR signaling pathway	Li et al., 2016
MicroRNA	MicroRNA-122	Its knockdown induces PTEN-PI3K-Akt signaling-mediated autophagy	Zhang et al., 2017
	MicroRNA-30a	Transferred through exosome	Yang et al., 2016
Others	Metformin	Inducing AMPK-mediated autophagy	Paneni et al., 2015
	Berberine	Activating p38 MAPK inhibition and phosphor-Akt activation	Zhang et al., 2014
	Rapamycin	Suppressing NF-κB-mediated inflammatory reaction	Chen et al., 2013; Wu et al., 2014
	Ginkgolide K	Enhancing IRE1α/X XBP1 activity Increasing ER-associated degradation-mediated clearance of misfolded proteins	Wang et al., 2016
	Exercise	Reducing mitochondrial number/size ratio Increasing mitochondrial bioenergetics	Tao et al., 2015; Campos et al., 2017

### Apoptosis Inhibitors

During acute myocardial infarction, apoptosis is widely considered to be involved in a large number of cardiomyocyte death as well as progressive loss of surviving cells in failing hearts (Takemura and Fujiwara, 2006). As a result, suppressing apoptosis in cardiomyocytes provides a potential and effective strategy in the alleviation of acute myocardial infarction. So far, several autophagy inducers have been reported to be effective in alleviating acute myocardial infarction taking advantage of apoptosis inhibition. For example, Liu et al. (2017) demonstrated that Apelin, the endogenous ligand for the G-protein-coupled APJ receptor, could suppress cardiac apoptosis *via* enhancement of autophagy, thus significantly decreasing myocardial infarction size and alleviating myocardial ischemia/reperfusion injury. Those effects were associated with the activation of Apelin/APJ system. It was also shown by Li et al. (2017) that Tongxinluo, a traditional Chinese medicine, produced a cardioprotective role against acute myocardial infarction *via* attenuating apoptosis in cardiomyocytes by inducing AMPK-mediated autophagy. Furthermore, human cellular repressor of E1A-stimulated genes (CREG), a secreted glycoprotein that regulated tissue and cell homeostasis, was reported to attenuate cardiac fibrosis after ischemia/reperfusion injury through the inhibition of apoptosis and enhancement of autophagy *via* regulation of lysosomal protein transfer, indicating a potential protective effect of CREG in myocardial infarction (Song et al., 2017). In addition, it was demonstrated that atorvastatin was involved in the regulation of apoptosis and autophagy process *via* the AMPK-mTOR signaling pathway, thus producing a cardioprotective role during acute myocardial infarction (Li et al., 2016).

### MicroRNAs

MicroRNAs refer to small non-coding RNA molecules functioning in RNA silencing and post-transcriptional regulation of gene expression (Ambros, 2004). In acute myocardial infarction, an increasing number of microRNAs have been reported to be useful both as biomarkers for heart injury detection and therapeutics to overcome limitations of past strategies and treat the lesions (Paiva and Agbulut, 2017). Among several of them, their functions in acute myocardial infarction are involved in the induction of cardiac autophagy. For example, Zhang et al. (2017) demonstrated that knockdown of microRNA-122 protected cardiomyocytes against hypoxia injury *via* the induction of chromosome 10 (PTEN)-PI3K-Akt signaling-mediated autophagy, indicating that targeting microRNA-122 might be a potential therapeutic strategy in the treatment of acute myocardial infarction. Another microRNA, microRNA-30a, was also reported to be detrimental in the induction of the protective autophagy in cardiomyocytes under hypoxia, transferred through the secretion of exosomes in the serum of acute myocardial infarction patients, suggesting the therapeutic role of inhibiting microRNA-30a in acute myocardial infarction (Yang et al., 2016).

### Others

Besides those two classifications described above, several other autophagy inducers have opened up in the treatment of acute myocardial infarction. It has been reported that metformin, a biguanide often used in the treatment of diabetes, produced a favorable effect on left ventricular function after acute myocardial infarction regardless of glycemic control (Paneni et al., 2015). This cardioprotective effect was investigated to be involved in the induction of AMPK-mediated autophagy process (Paneni et al., 2015). In addition, berberine, a quaternary ammonium salt from the protoberberine group of benzylisoquinoline alkaloids, was shown to attenuate adverse left ventricular remodeling and improve cardiac function in acute myocardial infarction animal models through autophagy induction mediated by the activation of p38 MAPK inhibition and phosphor-Akt activation (Zhang et al., 2014).

Furthermore, rapamycin, a classic autophagy inducer, was also reported to contribute to the attenuation of cardiac remodeling and dysfunction after acute myocardial infarction through the suppression of the overactivated NF-κB-mediated inflammatory cascade, since the occurrence of acute myocardial infarction was reported to contribute to the overwhelming induction of inflammatory reaction (Wu et al., 2014). This cardioprotective effect was further proven by fluorescence molecular tomography in acute myocardial infarction patients (Chen et al., 2013). Another autophagy inducer, ginkgolide K, was demonstrated to reduce infarct size, rescue heart dysfunction, and ameliorate endoplasmic reticulum (ER) dilation through the enhancement of inositol-requiring enzyme 1α (IRE1α)/X box-binding protein-1 (XBP1) activity and increase of ER-associated degradation-mediated clearance of misfolded proteins and autophagy (Wang et al., 2016).

Besides those autophagy inducing agents, it is interesting to prove that taking exercise contributed to the attenuation of acute myocardial infarction through the improvement of cardiac autophagy flux (Campos et al., 2017). This protective effect was led to by reducing the mitochondrial number/size ratio as well as increasing mitochondrial bioenergetics and better cardiac function (Campos et al., 2017). As a result, taking exercise training was regarded as a potential and effective therapy against acute myocardial infarction (Tao et al., 2015).

## Conclusion

**Figure 1:**
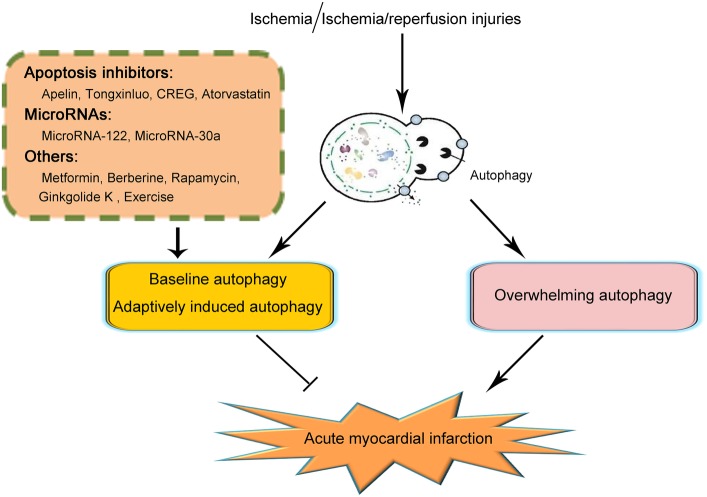
Schematic illustration of the role of autophagy in acute myocardial infarction. On the occurrence of acute myocardial infarction, cardiomyocytes suffered from ischemic or ischemia/reperfusion injury. Baseline or adaptively induced autophagy contributes to the alleviation of acute myocardial infarction. However, overwhelming induction of autophagy plays a detrimental role in acute myocardial infarction. So far, several clarifications of agents or pathways taking advantage of maintaining the function of baseline autophagy or adaptively inducing autophagy have been reported to be effective in the alleviation of acute myocardial infarction. Those agents or pathways include several apoptosis inhibitors such as Apelin, Tongxinluo, CREG, and Atorvastatin; microRNAs including MicroRNA-122 and MicroRNA-30a; and other agents including metformin, berberine, rapamycin, ginkgolide K, and exercise. Because of the complication of the effects and mechanisms of autophagy in acute myocardial infarction, the specific pathways in taking advantage of autophagy to effectively attenuate acute myocardial infarction remain unclarified. Further studies are demanded on this issue.

The past decade has witnessed the increasing understanding of the biology of autophagy and its roles in various kinds of disorders. Here, we reviewed both the protective and detrimental effects of autophagy in the pathogenesis and progression of acute myocardial infarction under ischemic or ischemia/reperfusion injuries (illustrated in Figure 1). We demonstrated that baseline autophagy or adaptively induced autophagy contributed to the alleviation of ischemic or ischemia/reperfusion damage while overwhelmingly induction of autophagy was detrimental during acute myocardial infarction. In addition, several agents and therapeutics for the treatment of acute myocardial infarction taking advantage of autophagy were also summarized. Based on the previous studies on the issue of autophagy in acute myocardial infarction, so far, several strategies could be made in the regulation of autophagy induction, including controlling the doses of autophagy inducers and monitoring cardiac functions when applying agents taking advantage of autophagy or elution on stents after coronary angioplasty. However, since the mechanisms of autophagy in acute myocardial infarction are complicated, so far, no specific pathway through which autophagy could be properly induced to be protective in acute myocardial infarction while getting rid of the detrimental effects of autophagy was elucidated. As a result, to ultimately take advantage of autophagy in the treatment of autophagy, further studies are demanded.

## Author Contributions

DW and KZ analyzed concerned literatures and wrote the manuscript. PH revised the manuscript. All the authors agreed to be accountable for the content of the work.

### Conflict of Interest Statement

The authors declare that the research was conducted in the absence of any commercial or financial relationships that could be construed as a potential conflict of interest.
